# Analysis of serum fatty acid, amino acid, and organic acid profiles in gestational hypertension and gestational diabetes mellitus *via* targeted metabolomics

**DOI:** 10.3389/fnut.2022.974902

**Published:** 2022-08-26

**Authors:** Xiangju Kong, Qiushuang Zhu, Yuanjie Dong, Yuqiao Li, Jinxiao Liu, Qingna Yan, Mingli Huang, Yucun Niu

**Affiliations:** ^1^Department of Gynaecology, The First Affiliated Hospital of Harbin Medical University, Harbin, China; ^2^Department of Nutrition and Food Hygiene, Public Health College, Harbin Medical University, Harbin, China

**Keywords:** hypertensive disorders of pregnancy, gestational diabetes mellitus, metabolomics, fatty acid, amino acid, organic acid

## Abstract

This study aimed to characterize metabolite differences and correlations between hypertensive disorders of pregnancy (HP) and gestational diabetes mellitus (GDM) using univariate, multivariate analyses, RF, and pathway analyses in a cross-sectional study. Dietary surveys were collected and targeted metabolomics was applied to measure levels of serum fatty acids, amino acids, and organic acids in 90 pregnant women at 24–28 weeks gestation at the First Affiliated Hospital of Harbin Medical University. Principal components analysis (PCA) and partial least squares-discriminatory analysis (PLS-DA) models were established to distinguish HP, GDM, and healthy, pregnant control individuals. Univariate and multivariate statistical analyses and Random Forest (RF) were used to identify and map co-metabolites to corresponding pathways in the disease states. Finally, risk factors for the disease were assessed by receiver operating characteristics (ROC) analysis. Dietary survey results showed that HP and GDM patients consumed a high-energy diet and the latter also consumed a high-carbohydrate and high-fat diet. Univariate analysis of clinical indices revealed HP and GDM patients had glycolipid disorders, with the former possessing more severe organ dysfunction. Subsequently, co-areas with significant differences identified by basic discriminant analyses and RF revealed lower levels of pyroglutamic acid and higher levels of 2-hydroxybutyric acid and glutamic acid in the GDM group. The number of metabolites increased in the HP group as compared to the healthy pregnant control group, including pyroglutamic acid, γ-aminobutyric acid (GABA), glutamic acid, oleic acid (C18:1), and palmitic acid (C16:0). ROC curves indicated that area under curve (AUC) for pyroglutamic acid in the GDM group was 0.962 (95% CI, 0.920–1.000), and the AUC of joint indicators, including pyroglutamic acid and GABA, in the HP group was 0.972 (95% CI, 0.938–1.000). Collectively, these results show that both GDM and HP patients at mid-gestation possessed dysregulated glucose and lipid metabolism, which may trigger oxidative stress *via* glutathione metabolism and biosynthesis of unsaturated fatty acids.

## Introduction

Gestational diabetes mellitus (GDM) refers to various degrees of abnormal glucose metabolism that occur during pregnancy, even though not developing type 2 diabetes mellitus (T2DM). It is one of the most common pregnancy complications in clinical practice. The incidence of GDM in the world is reported to be 1–14% and 1–5% in China, and there has been a significant upward trend in incidence in recent years (1). Prior studies have shown that GDM usually disappears after delivery, but women with a history of GDM have a sevenfold higher risk of developing T2DM (2). The main physiological mechanisms that contribute to GDM have increased insulin resistance and decreased insulin secretion (3). Yet, controversies exist regarding the etiology, risk factors, pathogenesis, and diagnostic criteria of GDM.

Regarding hypertensive disorders of pregnancy (preeclampsia or gestational hypertension), a group of diseases that coexist with pregnancy also increase blood pressure and are the primary factors contributing to maternal and perinatal mortality. It has been estimated that preeclampsia complicates 2–8% of pregnancies globally (4). So far, preeclampsia is mainly identified by measuring blood pressure during prenatal care in the international community. Although medical mercury sphygmomanometers are relatively accurate, it is difficult for a single person to use. Moreover, up to 50% of automatic oscillometric electronic sphygmomanometers must be calibrated every 6 months, resulting in calibration drift and inaccurate blood pressure readings over time between calibration periods (5–7). Therefore, the American College of Obstetrics and Gynecology (ACOG) emphasizes that risk evaluation regarding hypertensive disorders complicating pregnancy is crucial to the early prevention and treatment of these disorders. They also recommend screening for preeclampsia *via* maternal risk factors.

Women who develop GDM and HP are more likely to be obese and develop insulin resistance, endothelial dysfunction, oxidative stress, and metabolic disorders (8–11). GDM and HP are also risk factors for the future development of cardiovascular disease. Darcy et al. showed that women who develop preeclampsia have a higher risk of future cardiovascular disease and diabetes compared to women who have uncomplicated pregnancies (12). Evidence also suggests that preeclampsia is at least partially mediated by insulin resistance and that individuals with preeclampsia may have clinically silent but persistent alterations in insulin resistance (13). Moreover, a previous retrospective pilot study showed that falling insulin requirements is a possible sign of preeclampsia. Although counterintuitive, it is not uncommon for women to have a paradoxical drop in their insulin requirement in late pregnancy (14). Because both GDM and HP are common complications of pregnancy, they may follow similar metabolic trajectories.

A prospective nested case-control study in Chinese women suggested lipids play important biological functions and the associations between specific lipid species and GDM were partially explained by glycemic and insulin-related indicators (15). Several studies discovered that metabolic dysregulation, amino acid (AA) and organic acid (OA) dysmetabolism, were present years before diabetes onset among women with GDM (16–18). Most studies in the field of GDM and HP have only focused on one disease area or a single metabolic profile, as mentioned above.

So, in addition to observing the metabolic differences with OA, free fatty acid (FFA), and AA profiles examined, we also explored whether GDM and HP shared similarities in metabolite profiles and whether the metabolic pathways affected in GDM and HP intersect. Identifying and assessing the potential alterations of GDM and HP may further enlighten possible etiologies and pathogenesis underlying these peripartum morbidities.

## Materials and methods

### Design and study population

A cross-sectional study design including participants treated during the same period at the First Affiliated Hospital of Harbin Medical University was employed. Hypertensive disorders of pregnancy included gestational hypertension (GH) and pre-eclampsia (PE). GH was defined as systolic blood pressure (SBP) ≥ 140 mmHg and/or diastolic blood pressure (DBP) ≥ 90 mmHg, as assessed by the same nurse at the hospital at two or more separate visits between 24 and 28 weeks gestation. PE was defined as GH combined with proteinuria (19). GDM was defined as either a fasting plasma glucose (FPG) > 5.1 mmol/L, 1 h-PG > 10.0 mmol/L, or 2 h-PG > 8.5 mmol/L (20). Exclusion criteria included cases in which there was pre-existing diabetes, a family history of type 2 diabetes and hypertension, multifetal gestation, spontaneous abortion, malignant tumors, organ transplant, or the occurrence of acute complications, such as diabetic ketoacidosis or severe heart, liver, or kidney damage, etc. Women treated with hormones or immunosuppressive therapy before or during the first 3 months of pregnancy were also excluded, as well as patients taking medications that may affect glucose metabolism. Any patient diagnosed with GDM combined with HP was excluded from the study. Finally, 90 pregnant women (aged 20–40 years) with complete basic obstetrics and gynecology department data were admitted to the study as the final analytic population and included 30 controls, 30 GDM cases, and 30 HP cases. All selected candidates received oral and written information about the study and signed an informed consent to publish this paper. The research was conducted with the permission of the Ethics Committee of Harbin Medical University and is registered at www.chictr.org.cn as ChiCTR1900027669.

### Data and sample collection

Three treatment groups were included in the study: normal control pregnancy (NC), GDM, and HP, with 30 women in each group. Age, body mass index (BMI), gestational week, reproductive history, such as parity and abortion, biochemical laboratory indicators, and diagnostic information (GDM and HP) were collected for each participant. A 2-h 75-g oral glucose tolerance test (OGTT) was performed for all participants at 24–28 weeks gestation, and, at the same time, a food-frequency questionnaire (FFQ) was administered face-to-face. All participants were followed until the pregnancy due date. Fasting serum samples were collected only once at the time of recruitment for blood biochemical tests and targeted metabolomics. The serum samples collected were centrifuged at 3,000 rpm for 10 min at room temperature and stored at –80°C until metabolomics analysis. All pregnant women accepted clinical evaluation near the pregnancy due date.

### Targeted metabolomics assays

See Supplementary material for serum pretreatment. For organic acid and free-fatty acid detection with gas chromatography-mass spectrometry (GC-MS), chromatographic conditions were tested using the TRACE1310 gas chromatograph and the TSQ9000Evo mass spectrometer (Thermo Finnigan, Austin, TX, United States). The column used was the capillary TG-WAX (30 m × 0.25 mm, 0.25 μm film thickness). For amino acid detection with ultra-performance liquid chromatography-mass spectrometry (UPLC-TQ-MS), the ACQUITYTM UPLC system (Waters Corporation, Milford, CT, United States) was used with a HILIC column (100 mm × 2.1 mm × 1.7 μm, Waters Corporation, Milford, CT, United States). In the above three tests, the quality control (QC) sample was a mixture of equal volumes of samples to be tested. One QC sample was injected at every tenth sample injection to control for batch effects.

### Identification of remarkable metabolites by partial least squares-discriminatory analysis and orthogonal partial least squares-discriminatory analysis

Seventy-two metabolites, including 29 organic acids, 16 free fatty acids, and 27 amino acids, whose intra- and inter-coefficients of variation were less than 30%, were quantified. Metabolite results were normalized (by *Z*-score) and processed, and PCA, PLS-DA, and OPLS-DA were performed for basic discriminant analysis. First, PCA in unsupervised analysis mode was used to observe any separation effect within all samples, especially within QC samples. Then PLS-DA and OPLS-DA were carried out with 72 metabolites as X variables and 90 serum samples as Y variables, and modeling parameters R2Y and *Q*2 were used to evaluate the model. To avoid over-fitting of the model, the variables of the classification Y matrix that were defined when the model was established were randomly arranged 200 times, and the random different corresponding *Q*2 values were considered as the standard to measure whether the model was over-fitted. If *Q*2 was on the right side of the random distribution and *p* < 0.01, the OPLS-DA model was considered to have good stability and good predictability. OPLS-DA can also distinguish the response variables that most significantly contribute to the model. Thus, the variable importance in projection (VIP) value was calculated, and a VIP > 1.0 was used as subsequent screening criteria. Eventually, three statistical criteria were applied to identify the unique metabolites related to GDM and HP, namely, fold change (FC) of metabolites between GDM or HP versus NC (FC > 1.20 or FC < 0.83), adjusted *p*-value (*p* < 0.05) and the VIP value (VIP > 1.0).

### Identification of remarkable metabolites by random forest analysis

The random forest algorithm is a combination of bagging and a decision tree. Training samples were randomly selected using the Bootstrapping method to obtain decision trees. “Mean Decrease Accuracy” and “Mean Decrease Gini” were used to measure the importance of a metabolite in discriminant grouping in RF. If the value of a metabolite was changed into a random number, the prediction accuracy of RF was defined as the “Mean Decrease in Accuracy.” “Mean Decrease Gini” is the effect of a metabolite on the heterogeneity of observed values at all nodes of the classification tree. The greater the two values, the greater the importance of the metabolite in the random forest.

### Correlation analysis and pathway analysis

Correlation analysis was conducted by comparing these specific and critical metabolite correlations and observing whether metabolites generated change linking to clinical biochemical indicators. To further clarify the most relevant metabolic pathways in GDM and HP, pathway-enrichment analysis and pathway-topology analysis were performed. The most influential matched metabolic pathway had a pathway impact > 0.02, based on the pathway topology analysis, and -log10 (*p*) > 1.5, as determined by the pathway enrichment analysis.

### Risk assessment by receiver operating characteristics analysis

Receiver operating characteristics curve analysis can easily check the ability to identify diseases at any threshold value of each metabolite. Hence, the shared and distinct metabolites from two diseases were used to construct a binary logistic regression model in the SPSS software, and then the ROC curve was generated using the R software to evaluate the ability of shared metabolites to act as indicators for risk assessment.

### Statistical analyses

Qualitative variables are expressed in the form of categorical variables. Serum biochemical indices and metabolites are shown as mean ± SD or median and quartile. One-way ANOVA and Kruskal–Wallis tests were used to compare normally distributed data and non-parametric numerical data between groups. The Chi-square test was used to compare categorical variables between the three groups. False Discovery Rate (FDR) was used for multiple comparisons correction. SPSS statistical software (version 25.0) was used for univariate analysis. Targeted metabolites concentrations were analyzed by multivariate analysis using the SIMCA-P 14.1 software (Umetrics, Umeå, Sweden). RF correlation analysis and ROC analysis were performed by the R software (version 4.0.2). And then, pathway analysis was performed by Metaboanalyst 5.0.^1^ Spearman’s correlation coefficient was calculated for correlation analysis. Significance was set at *p* < 0.05.

## Results

### Study participant characteristics

Age, abortion frequency, and fertility frequency among the three groups were similar (Table 1). Compared with the NC group, BMI, blood glucose, LDL-C, HDL/LDL ratio, and triglycerides (TG) in the GDM group, and GLU, SBP, and DBP in the HP group were significantly increased (*p* < 0.05). The HP group had earlier gestational weeks compared to the NC and GDM group (*p* < 0.05). Beyond that, several biochemical parameters indicating viscera function (ALT, GGT, LDH, HBDH, etc.) in the HP group were also significantly higher than in the NC and GDM groups (Table 1). According to these findings, HP and GDM may involve a large-scale multisystem disturbance with adverse maternal outcomes, with HP having a greater deleterious effect. Additionally, fat intake, carbohydrate intake, and protein intake in 90 participants are presented in Supplementary Table 4. Dietary survey results showed that HP and GDM patients consumed high energy diets and that GDM consumed a high-carbohydrate and a high-fat diet (Table 1).

**TABLE 1:** Anthropometric and clinical characteristics of 90 subjects.

Parameter	NC	GDM	HP
Case, *n*	30	30	30
Age, years	29.83 ± 3.68	29.23 ± 2.87	29.53 ± 2.30
BMI, kg/m^2^	27.19 ± 3.02	29.10 ± 3.69*	28.50 ± 2.20
Gestational weeks, w	38 (38–39)	39 (38–39)	37 (36–37)**^#^※**
Abortion frequency, *n*%			
0	21 (70.00)	25 (83.33)	22 (73.33)
1	5 (16.67)	4 (13.33)	5 (16.67)
2	3 (10.00)	1 (3.33)	2 (6.67)
3	1 (3.33)	0 (0.00)	1 (3.33)
Fertility frequency, *n*%			
0	22 (73.33)	24 (80.00)	18 (60.00)
1	7 (23.33)	6 (20.00)	10 (33.33)
2	1 (3.33)	0 (0.00)	2 (6.67)
Total energy intake, kcal	2852.37 ± 764.37	3696.83 ± 771.59*	3272.07 ± 463.06**^#^※**
Carbohydrate, kcal%	0.63 ± 0.12	0.56 ± 0.13*	0.62 ± 0.09**^※^**
Protein, kcal%	0.11 (0.09–0.16)	0.12 (0.08–0.16)	0.12 (0.10–0.16)
Fat, kcal%	0.23 (0.15–0.32)	0.29 (0.25–0.41)*	0.24 (0.19–0.30)
FPG, mmol/L	4.06 (3.86–4.56)	5.65 (5.30–6.21)*	4.48 (4.28–4.83) **^※^**
1h-PG, mmol/L	7.28 ± 1.17	10.08 ± 1.07*	7.50 ± 0.94**^※^**
2h-PG, mmol/L	6.66 ± 1.00	9.17 ± 0.99*	6.67 ± 1.38**^※^**
GLU, mmol/L	4.25 (4.03–4.43)	5.28 (4.95–5.55)*	4.59 (4.38–5.19)**^#※^**
SBP, mmHg	109.50 (104.75–115.50)	115.00 (109.50–119.25)	159.00 (148.75–172.00)**^#※^**
DBP, mmHg	74.50 (72.00–78.25)	77.50 (73.00–83.00)	110.00 (101.75–118.50)**^#※^**
HDL-C, mmol/L	2.03 ± 0.28	1.78 ± 0.33*	1.91 ± 0.48
LDL-C, mmol/L	2.12 (1.73–2.54)	2.95 (2.55–4.04)*	3.04 (1.68–3.36)
HDL/LDL	0.95 (0.70–1.31)	0.56 (0.47–0.69)*	0.72 (0.54–1.07)
CHOL, mmol/L	6.23 (5.25–7.11)	6.80 (5.96–8.26)	6.65 (6.23–7.08)
TG, mmol/L	3.69 (2.98–4.17)	4.75 (3.66–6.01)*	4.29 (3.58–5.07)
VLDL, mmol/L	0.75 (0.60–0.86)	0.96 (0.69–1.11)	0.91 (0.57–1.27)
ALT, U/L	8.75 (5.95–11.20)	9.85 (8.48–12.68)	12.15 (9.00–15.53)**^#^**
GGT, U/L	9.85 (8.05–11.55)	10.75 (8.80–12.85)	26.20 (14.13–36.65)**^#※^**
LDH, U/L	188.05 (149.50–214.75)	188.00 (158.50–228.00)	284.50 (240.50–334.50)**^#※^**
HBDH, U/L	117.00 (103.79–130.91)	115.00 (102.50–127.25)	159.00 (133.50–192.00)**^#※^**
BUN, mmol/L	2.85 (2.56–3.28)	3.00 (2.39–3.94)	4.43 (3.67–4.88)**^#※^**
Cr, mmol/L	43.90 (40.95–46.58)	46.25 (39.73–50.65)	53.86 (45.39–65.63)**^#※^**
UA, μmol/L	275.35 (247.00–315.55)	292.20 (239.58–365.20)	393.70 (329.77–480.35)**^#※^**

Data are shown as mean ± SD or median and quartile. False discovery rate was automatically corrected for multiple comparisons during Kruskal–Wallis tests. *p < 0.05 NC vs. GDM; ^#^*p* < 0.05 NC vs. HP;  ^※^*p* < 0.05 GDM vs. HP. BMI, body mass index; Gestational weeks, the average weeks of delivery for each group; FPG, fast plasma glucose; 1h-PG, 1h-plasma glucose from OGTT; 2 h-PG, 2 h-plasma glucose from OGTT; GLU, glucose; SBP, systolic blood pressure; DBP, diastolic blood pressure; HDL-C, high-density lipoprotein cholesterol; LDL-C, low-density lipoprotein cholesterol; CHOL, cholesterol; TG, triglyceride; VLDL, very low density lipoprotein; ALT, alanine aminotransferase; GGT, γ-glutamyl transferase; LDH, lactate dehydrogenase; HBDH, hydroxybutyrate dehydrogenase; BUN, blood urea nitrogen; Cr, creatinine; UA, uric acid.

### Univariate analysis for metabolites

**FIGURE 1:**
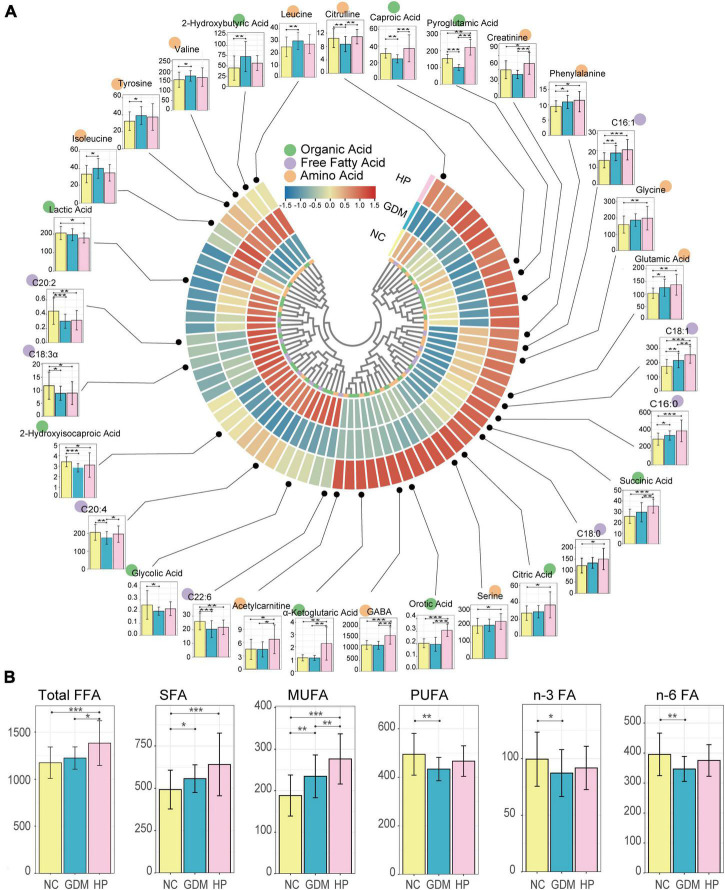
Differential distribution of serum metabolites in three groups. **(A)** Overall sample clustering heatmap showing the abundance of all targeted metabolites in different groups. In total, 72 metabolomic signatures are shown in a categorical tree, grouped as organic acid (green), free fatty acid (purple), and amino acid (orange) in the innermost circle. The outer circles show metabolites for which the mean abundances were markedly elevated (red) or decreased (blue) between NC, GDM, and HP groups. Box plots depict the abundance of various metabolites that were significantly (*p* < 0.05) different compared to NC (yellow bar). The *y*-axis for each box plot is abundance. Each plot shows boxes in the NC (left-most bar) and other two groups (GDM and HP) in order from left to right. **(B)** Box plots illustrating the distribution of serum free fatty acids in the NC, GDM, and HP groups. Total FFA, total fatty acid; SFA, saturated fatty acid; MUFA, monounsaturated fatty acid; PUFA, polyunsaturated fatty acid; n-3 FA, n-3 fatty acid; n-6 FA, n-6 fatty acid. *, **, and *** for *p* < 0.05, *p* < 0.01, and *p* < 0.001, respectively.

The overall sample clustering heatmap shows the abundance of 72 metabolites between groups, with the largest differences seen in the HP group (Figure 1A). As detailed, the OA (Supplementary Table 1), FFA (Supplementary Table 2), and AA (Supplementary Table 3) profiles are presented in three tables with actual measured values. As expected, metabolite distribution in HP was consistent with the results of the measured biochemical indicators, indicating that, compared with GDM, more serious metabolic dysfunction occurs in HP patients. Univariate analysis shows altered serum free fatty acids, amino acids, and organic acids in GDM and HP at mid-gestation compared with NC. Compared with the NC group, saturated fatty acids and monounsaturated fatty acids were significantly increased in the GDM group, and total fatty acids, saturated fatty acids, and monounsaturated fatty acids were significantly increased in the HP group. However, polyunsaturated fatty acids, n-3 fatty acids, and n-6 fatty acids were significantly decreased in the GDM group compared with the control group (*p* < 0.05). Compared with the GDM group, total fatty acids and monounsaturated fatty acids in the HP group were significantly increased (*p* < 0.05) (Figure 1B).

### Typical metabolic profiles revealed by partial least squares-discriminatory analysis and OPLS-DA

**FIGURE 2:**
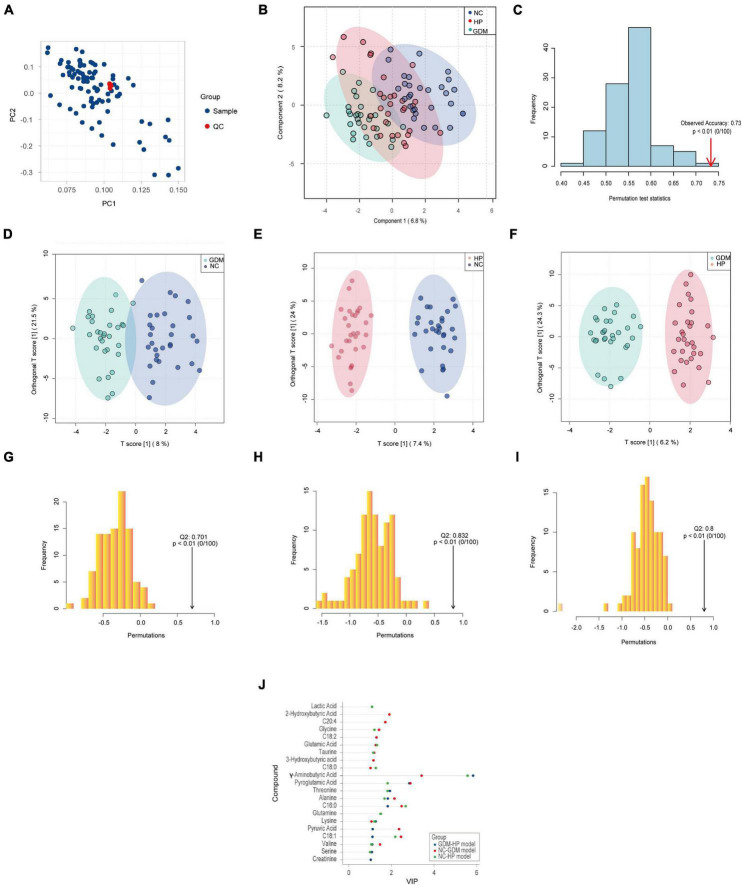
Typical metabolic profiles revealed by PLS-DA and OPLS-DA. **(A)** PCA analysis of 90 samples with QC. **(B)** PLS-DA analysis among three groups, with R2Y (cum) = 0.802 and *Q*2 (cum) = 0.725. **(C)** Permutation tests of PLS-DA discrimination models among three groups. **(D)** The score plots of the OPLS-DA model between NC and GDM, with R2Y (cum) = 0.812 and *Q*2 (cum) = 0.701. **(E)** The score plots of the OPLS-DA model between NC and HP, with R2Y (cum) = 0.948 and *Q*2 (cum) = 0.832. **(F)** The score plots of the OPLS-DA model between GDM and HP, with R2Y (cum) = 0.923 and *Q*2 (cum) = 0.800. Permutation tests of OPLS-DA discrimination models between NC and GDM **(G)** between NC and HP **(H)** between GDM and HP and **(I)** with the *Q*2 on the right side of the random distribution and *p* < 0.01, which indicated the above three OPLS-DA models were reliable and there was no over-fitting phenomenon. **(J)** VIP values of three OPLS-DA models.

To investigate whether GDM and HP could be identified based on metabolic disturbances, serum changes in GDM and HP were compared with NC. PCA analysis displayed that QC samples clustered well with no “outlier sample point” *via* DModx’s diagnosis (Figure 2A). The three groups were evident based on clustering generated by PLS-DA and OPLS-DA analyses, which showed that the metabolite composition and structure of the three groups were different (Figures 2B,D–F). Permutation tests suggested that the PLS-DA and OPLS-DA models were reliable, and there was no over-fitting phenomenon (Figures 2C,G–I). VIP values of the three OPLS-DA models only displayed metabolites with values greater than 1.0 (Figure 2J). Ultimately, C18:1, 2-hydroxybutyric acid, pyroglutamic acid, and glutamic acid in GDM were initially screened out, based on three statistical standards of FC between groups (FC > 1.20 or FC < 0.83), adjusted *p*-value (*p* < 0.05), and VIP value (VIP > 1.0). Simultaneously, C16:0, C18:0, C18:1, pyroglutamic acid, glycine, GABA, and glutamic acid, as distinct and critical metabolites, were mapped in the trajectory of HP (Table 2).

**TABLE 2:** Potential biomarkers discovered by VIP, FC, and adjusted *p*-values.

Different metabolites	VIP	FC	*P-value*
**NC vs. GDM**			
C18:1	2.45	1.24	**0.003**
2-Hydroxybutyric acid	1.91	1.62	**0.001**
Pyroglutamic acid	2.89	0.65	**<0.001**
Glutamic acid	1.27	1.21	**0.042**
**NC vs. HP**			
C16:0	2.67	1.32	**<0.001**
C18:0	1.27	1.24	**0.011**
C18:1	2.18	1.47	**<0.001**
Pyroglutamic acid	1.82	1.44	**0.001**
Glycine	1.20	1.25	**0.008**
γ-Aminobutyric acid	5.57	1.36	**<0.001**
Glutamic acid	1.33	1.33	**0.001**
**GDM vs. HP**			
C18:1	1.11	1.21	**0.005**
Pyroglutamic acid	2.84	2.21	**<0.001**
γ-Aminobutyric acid	5.84	1.38	**<0.001**
Creatinine	1.03	1.48	**<0.001**

*P*-values in bold indicate that there are significant differences between the NC, GDM, and HP groups.

### Typical metabolic profiles revealed by random forest

**FIGURE 3:**
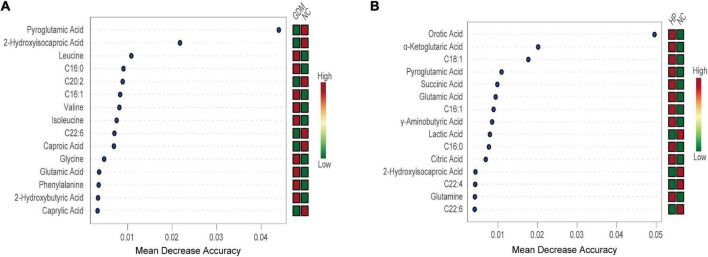
Random Forest revealed the 15 most important metabolites for NC vs. GDM **(A)** and for NC vs. HP **(B)**. Mean decrease accuracy is higher, and the impact of metabolites on GDM/HP is larger. The figure on the right is a heat map of the identified 15 metabolites in both groups. Red is high-expression; green is low-expression.

As presented in the RF results, 15 metabolites clearly achieved significance in GDM. These included decreased levels of pyroglutamic acid, 2-hydroxyisocaproic acid, C20:2, C22:6, caproic acid, and caprylic acid, and elevated levels of leucine, C16:0, C16:1, valine, isoleucine, glycine, glutamic acid, phenylalanine, and 2-hydroxybutyric acid (Figure 3A). These metabolites revealed a highly unique metabolic GDM phenotype, characterized by altered glutathione metabolism (pathway impact > 0.1, FDR < 0.05). Likewise, 15 of the most significant metabolites were identified in HP, including elevated levels of orotic acid, α-ketoglutaric acid, C18:1, pyroglutamic acid, succinic acid, glutamic acid, C16:1, GABA, C16:0, citric acid, and glutamine and decreased levels of lactic acid, 2-hydroxyisocaproic acid, C22:4, and C22:6 (Figure 3B). These metabolites revealed a highly unique metabolic HP phenotype, characterized by alterations in alanine, aspartate, and glutamate metabolism, butanoate metabolism, D-glutamine, and D-glutamate metabolism, arginine biosynthesis, and the citrate cycle (TCA cycle) (pathway impact > 0.1, FDR < 0.05).

### Shared metabolites with basic discriminant analysis and random forest analysis

**FIGURE 4:**
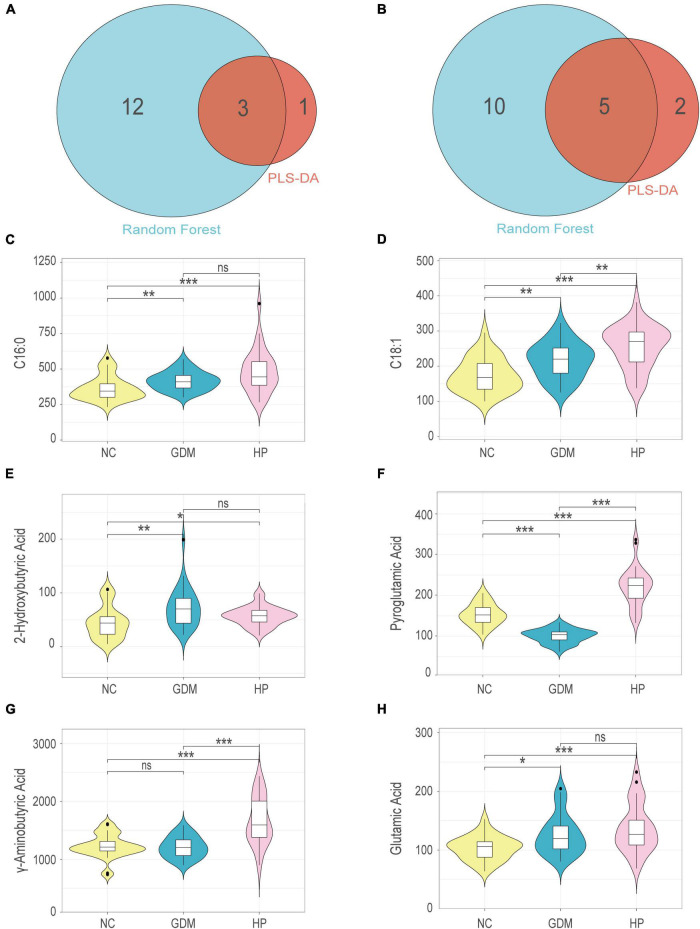
Venn diagram for shared and distinct metabolites screened out *via* basic discriminant analysis and random forest analysis within NC-GDM **(A)** and NC-HP **(B)**. Univariate analysis for each comparison for: **(C)** C16:0; **(D)** C18:1; **(E)** 2-Hydroxybutyric acid; **(F)** Pyroglutamic acid; **(G)** γ-aminobutyric acid; **(H)** Glutamic acid. *, **, and *** for *p* < 0.05, *p* < 0.01, and *p* < 0.001, respectively; ns, no significant difference.

Common metabolites identified by both basic discriminant analysis and RF analysis in GDM included 2-hydroxybutyric acid, pyroglutamic acid, and glutamic acid (Figure 4A). In HP, these included C16:0, C18:1, pyroglutamic acid, GABA, and glutamic acid (Figure 4B). Six expression profiles were violin-plotted to obtain representative differences in metabolites by one-dimensional statistical analysis. Compared with the NC group, 2-hydroxybutyric acid and glutamic acid were significantly increased in the GDM group, and pyroglutamic acid was decreased (Figures 4E,F,H). In addition, C16:0, C18:1, pyroglutamic acid, GABA, and glutamic acid were significantly increased in the HP group compared with the NC group (Figures 4C,D,F–H). In terms of the non-normal distribution and high variability of GABA and glutamic acid, we performed another method, the Weighted Correlation Network Analysis (WGCNA) to find hub metabolites (21, 22). WGCNA result was in accordance with the results of multivariate analysis and RF analysis, which indicated that GABA, pyroglutamic acid, and glutamic acid were screened out as hub metabolites in HP (Supplementary Figure 1).

### Correlation analysis and metabolic pathway reprogramming in gestational diabetes mellitus and hypertensive disorders of pregnancy

**FIGURE 5:**
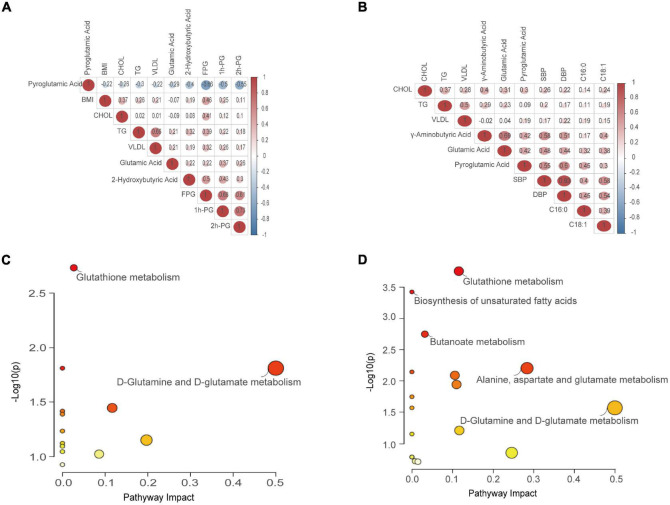
Correlation analysis and pathway enrichment analysis. **(A)** Correlation analysis between the screened differential metabolites and phenotypes referring to GDM. **(B)** Correlation analysis between the screened differential metabolites and phenotypes referring to HP. Values in red denote a positive trend, whereas those in green denote a negative trend. **(C)** Metabolic pathway reprogramming refers to GDM. **(D)** Metabolic pathway reprogramming refers to HP. Colors varying from yellow to red denote metabolites with differing levels of significance. Node shapes varying from small to large denote the number of metabolites included in a pathway.

To explore whether there is a cascade effect between the screened differential metabolites and disease phenotypes, correlation analysis was conducted. When comparing GDM and NC groups, pyroglutamic acid levels negatively correlated with BMI, CHOL, TG, VLDL, FPG, 1 h-PG, and 2 h-PG, with the negative correlation being most significant with FPG (*p* < 0.001). Conversely, 2-hydroxybutyric acid and glutamic acid were positively correlated with the above biochemical indicators. Additionally, glutamic acid positively correlated with TG/1h-PG, and 2-hydroxybutyric acid positively correlated with TG/FPG (*p* < 0.05) (Figure 5A). Interestingly, comparing HP and NC groups, five differential metabolites (C16:0, C18:1, pyroglutamic acid, GABA, and glutamic acid) positively correlated with SBP, DBP, CHOL, TG, and VLDL, especially strongly correlated with SBP (*p* < 0.05). Most encouragingly, GABA was moderately correlated with glutamic acid and pyroglutamic acid (*p* < 0.05) (Figure 5B). To further clarify the most relevant metabolic pathways in GDM and HP, pathway analysis showed the detailed impacts of GDM/HP-related alterations in metabolic networks. As labeled, two metabolic pathways were defined as disturbed in the serum profiles of GDM. These included glutathione metabolism and D-glutamine and D-glutamate metabolism (Figure 5C). Meanwhile, five metabolic pathways were defined as disturbed in the serum profiles of HP, including glutathione metabolism, D-glutamine, and D-glutamate metabolism, biosynthesis of unsaturated fatty acids, butanoate metabolism, and alanine, aspartate, and glutamate metabolism (Figure 5D).

### Risk assessment for gestational diabetes mellitus and hypertensive disorders of pregnancy

**FIGURE 6:**
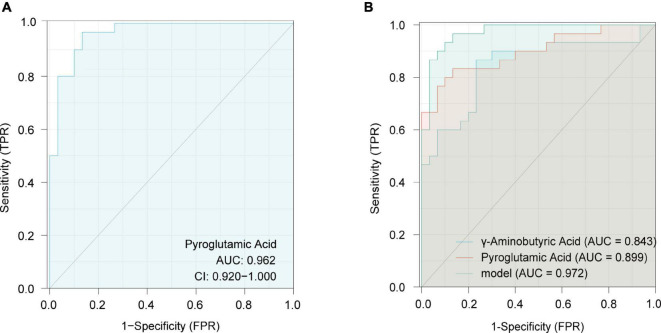
Receiver operating characteristics (ROC) curves in the GDM group for pyroglutamic acid **(A)** and in the HP group with joint indicators, including pyroglutamic acid and γ-aminobutyric acid **(B)**.

To reduce the false-positive risk in the metabolite selection procedure, binary logistic regression between NC and GDM/HP groups was conducted. Pyroglutamic acid (OR = 0.824, 95% CI, 0.720–0.944) remained a risk factor for GDM when using the NC-GDM model, and pyroglutamic acid (OR = 1.139, 95% CI, 1.014–1.279) and GABA (OR = 1.016, 95% CI, 1.000–1.033) performed similarly in HP using the NC-HP model, after adjusting for BMI as a confounding factor (Supplementary Table 5). Finally, pyroglutamic acid in GDM and pyroglutamic acid and GABA in HP were selected to construct logistic regression models, respectively. ROC analysis revealed that the AUC of pyroglutamic acid in the GDM group was 0.962 (95% CI, 0.920–1.000) (Figure 6A), and the AUC of joint indicators, including pyroglutamic acid and GABA, in HP group was 0.972 (95% CI, 0.938–1.000) (Figure 6B).

## Discussion

The baseline information revealed that the BMI in the GDM group was higher than in the NC. A meta-analysis published in *JAMA* reported that gestational weight gain exceeded weight gain recommended by the National Academy of Medicine in 47% of 130, 9136 pregnancies (23). Women with excessive weight gain during pregnancy are more prone to adverse maternal and post-partum outcomes (24). As such, a recent study aimed to confirm the impact of maternal BMI and glycemia on the fetal metabolome (25). Almost all obese GDM patients have increased levels of fatty acids (26), which was confirmed in this study. Additionally, LDL-C, CHOL, TG, and VLDL concentrations in the GDM group were notably increased compared with those of the NC group. A prior study showed that increased levels of fatty acids affect liver VLDL secretion by interfering with insulin action (27). On the one hand, fatty acids are synthesized into TG for incorporation into VLDL with the help of thioesterase superfamily member 2. On the other hand, thioesterase superfamily member 2 suppresses insulin signaling, inhibiting the degradation of apolipoprotein B in hepatocytes and stimulating microsomal TG transfer protein to increase VLDL secretion. In addition to these findings, the GDM and HP disease groups had a greater degree of dyslipidemia as compared with the NC group, with the HP group showing severely abnormal liver, heart, and kidney functions. This phenomenon has been seen in a prospective observational cardiovascular phenotypic study during mid-gestation in women who develop GDM and HP (28).

It is worth mentioning that the serum levels of 2-hydroxybutyric acid, glutamic acid, branched chain (leucine and isoleucine), and aromatic amino acids (tyrosine) in GDM women were notably higher, while pyroglutamic acid, caproic acid, 2-hydroxyisocaproic acid, glycolic acid, and citrulline were significantly lower than that in the NC group in this study. Specifically, increased concentrations of leucine and isoleucine generated higher levels of α-ketobutyric acid from cysteine metabolism related to oxidative stress, whose byproduct is 2-hydroxybutyric acid. Pyroglutamic acid is a cyclized derivative of glutamic acid, formed non-enzymatically from glutamate, glutamine, and γ-glutamylated peptides. In our study, decreased pyroglutamic acid levels were observed in GDM, demonstrating that pyroglutamic acid imbalances may be responsible for GDM occurrence. Furthermore, decreases in citrulline, a byproduct of the enzymatic production of nitric oxide from arginine, may be due to glutathione-induced inhibition of the eNOS pathway that leads to a decrease in NO bioavailability and metabolically induced cardiac dysfunction in GDM patients (29). It is interesting to note that the aforementioned metabolites were discovered to be the most significantly altered organic and amino acids that have been reproducibly confirmed to be clinical indicators of subclinical abnormalities in glucose metabolism. These metabolites also serve as biomarkers of type 2 diabetes (30–34). Thus, it appears that GDM and type 2 diabetes share similar metabolic reprogramming.

Early and late-onset preeclampsia has various pathophysiologic and etiologic pathways, including endothelial dysfunction, alterations in oxidative stress, metabolic disorder, and so on. In this study, elevated levels of pyroglutamic acid, glutamic acid, GABA, glycine, orotic acid, α-ketoglutaric acid, and succinic acid were observed in HP women. Although there are few studies on how these metabolites act within the pathological mechanisms of HP, they have been reported to play a role in glutathione metabolism that is upregulated by oxidative stress (35). A wealth of clinical studies have confirmed that glutathione synthesis is a biomarker of liver oxidative stress (36). Of these metabolites, α-ketoglutaric acid and succinic acid, a byproduct of glutamic acid and GABA, enter the TCA cycle. This may explain the increasing trend in blood glucose levels in HP patients at baseline, indicating that HP patients already possessed insulin resistance.

In addition, the proportion of saturated and unsaturated fatty acids was imbalanced in GDM and HP, mainly evidenced by increases in saturated fatty acid and monounsaturated fatty acid and the decline in polyunsaturated fatty acid. These alterations may be due to adipose tissue-specific insulin resistance and impaired glucose uptake in GDM, in which the body accelerates the oxidation and decomposition of fat for energy. Excessive production of fatty acids, in turn, may simultaneously act on other tissues to trigger insulin resistance. For instance, excessive saturated fatty acids stimulate endoplasmic reticulum stress, enhancing liver gluconeogenesis and insulin resistance (37). For HP, a systematic review revealed that free fatty acids spark endothelial dysfunction through several mechanisms, such as impaired insulin signaling transduction and nitric oxide production, oxidative stress, inflammation, activation of the renin–angiotensin system, and endothelial cell apoptosis (38). Moreover, elevated maternal serum lipids and LDL levels may induce endothelial dysfunction secondary to oxidative stress (39). Conversely, polyunsaturated fatty acids decreased the risk of GDM and HP and improve metabolic disorders. In fact, the latest research published in *Cell* showed that n-3 fatty acids control adipogenesis through ciliary signaling to enable the homeostasis of healthy fat tissue and ameliorate insulin resistance and metabolic dysfunction (40). Lou et al. found that n-3 fatty acid enrichment in the cell membrane, which increases the ratio of eicosapentaenoic acid to docosahexaenoic acid in the cell membrane, affected intracellular signal transduction and inhibited the transcriptional activity of the inflammation-related transcription factor NF-κB (41). The n-3 fatty acid also generates derivatives with anti-inflammatory, analgesic, and other physiological activities, such as resolvins and protectins, that stimulate neutrophils and macrophages to produce anti-inflammatory cytokines, including IL-4 and IL-10, and reduce the inflammatory response to regulate levels of inflammation (42).

Overall, comparative analysis, PLS-DA, RF, logistic regression, and pathway analyses were used to elucidate whether distinct metabolites changed during GDM and HP. Metabolic pathway analysis was employed to elucidate reprogramming pathways. Results showed almost a quarter of metabolites could be used to differentiate GDM and HP cases among healthy pregnant women. Of these, pyroglutamic acid and GABA, as co-metabolites, were associated with risk assessment for both diseases. Both GDM and HP showed dysregulation of glucose and lipid metabolism, which may trigger oxidative stress through alterations in glutathione metabolism and biosynthesis of unsaturated fatty acids. However, such expositions are unsatisfactory as the molecular pathogenesis of GDM and HP cannot be completely explained by these mechanisms, and we cannot determine the causality of the observed changes in metabolites in the present study. Thus, further animal and prospective population experiments are needed to explore the cause-and-effect relationships between metabolites alterations and disease onset and progression. Moreover, much of the research to date has been descriptive in nature, and the enrolled subjects do not represent a random sample of Chinese pregnant women. Collectively, these novel and intriguing metabolites characterized by metabolomics will facilitate greater awareness of GDM and HP, so that efficient strategies for disease prevention, antepartum maternal and fetal evaluation, and patient monitoring can be implemented.

## Data availability statement

The original contributions presented in this study are included in the article/Supplementary material, further inquiries can be directed to the corresponding authors.

## Ethics statement

The studies involving human participants were reviewed and approved by the Ethics Committee of Harbin Medical University. The participants provided their written informed consent to participate in this study. Written informed consent was obtained from the individual(s) for the publication of any potentially identifiable images or data included in this article.

## Author contributions

XK and QZ were involved in the protocol design and data analyses and drafted the manuscript. YD contributed to the conception and methodology. YL and MH participated in a questionnaire FFQ and follow-up visit. JL and QY conceived and designed the study protocol and created a visualization of the results. YN contributed to the funding acquisition and had primary responsibility for the final content. All authors contributed to the article and approved the submitted version.

## Footnotes

1
www.metaboanalyst.ca
