# Supplementary material for: Analysis of serum fatty acid, amino acid, and organic acid profiles in gestational hypertension and gestational diabetes mellitus via targeted metabolomics
Source: Front Nutr. 2022 Aug 26;9:974902. doi: 10.3389/fnut.2022.974902 (PMC9458889; doi:10.3389/fnut.2022.974902)
Supplement: Supplementary file 1 [file Data_Sheet_1.PDF]

Supplementary Material

1 Serum Pretreatment and Instrument Condition of Organic Acid, Free Fatty Acid and Amino acid

1.1 Organic acids detection with GC-MS

Serum samples (100 μL) were placed in an EP tube and 9 μL succinate-2,2,3, 3-D4 solution (100 g/mL) was added as internal standard. 300 μL methanol was added as the protein extraction reagent. Vortex-mixed for 4 min, and the supernatant was centrifuged at 14000 rpm/min at 4 °C for 15 min. The supernatant was extracted and centrifuged at 14000 rpm/min at 4 °C for 10min for a second time. Nitrogen was blown for 45 min until completely dry. Subsequently, 30 μL methoxide pyridine solution (33 mg/ml) was added. After vortexed for 1.5min, the product was placed in a constant temperature oscillator box at 180 r/min and oximation reaction was conducted at 37 °C for 1.5 h. 45 μL of MSTFA derivative reagent was added to the oxime-treated samples. After fully eddy, the samples were further set in a constant temperature oscillating chamber at 40 °C. Afterwards, oscillation at 180 r /min for 10 min, the samples were left for 1h, and the supernatant was transferred to the injection bottle. Per 2 μL supernatant preprocessed was transferred to the injection bottle for need.

Chromatographic conditions were tested using the TRACE1310 gas chromatograph and the TSQ9000Evo mass spectrometer (Thermo Finnigan, Austin, TX, USA). The TG-WAX capillary column (30 m × 0.25 mm, 0.25 μm film thickness) was used. The measured temperature was as follows: (1) the initial temperature was 70 °C, which was increased to 155 °C at a rate of 5 °C/min for 2 min; (2) increased to 170 °C at a rate of 5 °C/min and kept for 2 min; (3) increased to 220 °C at a rate of 5 °C /min for 2 min. Mass spectrometry detection conditions: Delivery line temperature maintained at 250 °C. The solvent delay time was 6 min. The source temperature was 250 °C, the electron energy was 70 eV, and the selective reaction was detected by SRM scanning.

1.2 Free-fatty acids detection with GC-MS

Serum samples were thawed in 4 ℃ and fully mixed about 20 s. A volume of 200 μL thawed serum was transferred into a 10 mL glass tube, then 200 μL internal standard solution (200 μg/mL heptadecanoic acid) and 2 mL KOH-CH3OH were added into each sample, vortex-mixed for 30 s and placed at room temperature for 10 min. A certain amount of anhydrous sodium sulfate and 1mL hexane was added into the test tube, vortex-mixed for 1 min. The solution was centrifuged at 3500 rpm for 5 min and the hexane layer was placed into a 4 mL Eppendorf tube. Then another 1 mL hexane was added into the same tube and extracted the hexane layer again based on the same operation. The collection was discarded. Subsequently, 2 mL 10% H_2SO_4-CH_3OH was added into the residuary serum, reacted at 62 °C water bath for 2 h. After the solution cooled to the room temperature, some anhydrous sodium sulfate was added to remove traces of water. After adding 2 mL hexane and mixing for 1 min, the hexane layer was collected and transferred to a new tube after centrifuging at 3500 rpm for 5 min. Then collection was evaporated to dryness under N2 by Bath Nitrogen Blow Instrument (TTL-DCI, Beijing, China) and reconstituted in 100 μL hexane. The

solution was centrifuged at 3500 rpm for 30 s and placed into a sampling vial pending for free fatty acids (FFA) analysis by GC-TQ-MS/MS.

For GC-TQ-MS/MS analysis, a TRACE 1310 gas chromatograph coupled with a TSQ 8000 Evo mass spectrometer (Thermo Finnigan, Austin, Texas, USA) was used. Helium was used as the carrier gas. A split injector (the split ratio being 1:10) was used to add the sample (1.0 μL) onto a Thermo TG-WAX MS (30 m×0.25 mm I.D., 0.25 μm film thickness) capillary column at 230 ℃. Free fatty acid methyl esters were separated in constant flow by the following oven program: (1) after the initial temperature is 50 ℃ for 1 min, the temperature was raised to 200 ℃ at the speed of 10 ℃/min and kept for 10 min; (2) raised the temperature to 220 ℃ at a speed of 5 ℃/min and kept it for 11 min. Mass spectrometry detection conditions: the temperature of the transmission line was maintained at 230 ℃. The solvent delay time was 5 min. The source temperature was 230 ℃ and the electron energy was 70eV. The triple quadrupole mass spectrometer was operated under electron impact ionization mode. The Mass spectra of m/z 30-450 was collected by multiple reaction monitoring for MS/MS measurement.

1.3 Amino acid detection with UPLC-TQ-MS

As usual, our sample needed pretreatment. We took the serum to be tested out of the refrigerator at －80 °C and put it into the refrigerator at 4 °C for melting. The serum and additives were vortexed, and 30 μL of the serum mixture was added into an EP tube with 10 μL (100 μg/mL) of stable isotope labeled valine-d8 and phenylalanine-d8 as a mixed internal standard solution. 80 μL of sample treatment solution (74.9% methanol, 24.9% acetonitrile, 0.2% formic acid), swirled for 30 s, and let it stand on ice for 30 min. Centrifuged (4 °C, 15000 rmp/min, 20 min), and the supernatant was put into a new EP tube. Overnight at- 20 °C (12 h), and the second centrifugation (4 °C, 15000 rmp/min, 15 min). Later the supernatant prepared at the inner liner tube was placed 2 μL of it into the liquid injection tray for detecting.

ACQUITYTM UPLC system (Waters Corporation, Mil-ford, USA) and the HILIC column (100 mm × 2.1 mm × 1.7 μm, Waters Corporation, Milford, USA) were prepared. The flow rate was 0.30 mL/min, and the injection volume per needle was 2 μL. The mobile phase A was 0.2% formic acid, 5% acetonitrile and 2.5 mmoL/L ammonium formate dissolved in water. The mobile phase B was 0.2% formic acid, 5% water and 2.5 mmoL/L ammonium formate dissolved in acetonitrile. Gradient elution procedure was as follows: (1) 5% A concentration for 0 - 0.5 min; (2) increased to 40% within 6 min; (3) increased to 50% within 6-7 min; (4) 50% A kept for 7-8 min, and balanced to initial conditions within 8-15 min. Mass spectrometry detection conditions: Waters Xevo TQD mass spectrometer (Waters Corporation, Manchester, UK) was used. The flow rate was 650 L/h and the cone gas flow rate was 50 L/h. The temperature of desolvation gas was 400 °C, and the temperature of ion source was 150 °C. The capillary voltage was 3200 V.

2 The OA, FFA and AA profiles of 90 pregnant women

2.1 OA profiles by GC-MS

TABLE S1 | The OA profiles of 90 subjects.

OA (μg/mL)	NC (n = 30)	GDM (n = 30)	HP (n = 30)
Pyruvic Acid	237.94 ± 125.60	192.09 ± 69.99	220.82 ± 93.03
Lactic Acid	205.85 ± 36.86	196.46 ± 32.52	178.72 ± 26.81
Caproic Acid	31.19 ± 5.56	24.70 ± 48.8	37.24 ± 16.16
Glycolic Acid	0.26 ± 0.12	0.20 ± 0.034	0.23 ± 0.58
2-Hydroxybutyric Acid	44.64 ± 29.57	72.50 ± 36.55	56.83 ± 17.80
Oxalic Acid	11.64 ± 1.14	11.51 ± 0.53	11.58 ± 1.32
3-Hydroxybutyric acid	39.66 ± 7.34	51.16 ± 21.25	42.24 ± 12.72
Malonic	1.79 ± 0.00	1.79 ± 0.00	1.80 ± 0.00
Methylmalonic Acid	0.96 ± 0.72	0.79 ± 0.61	1.30 ± 0.74
2-Hydroxyisocaproic Acid	3.41 ± 0.47	2.85 ± 0.40	3.10 ± 1.16
Caprylic Acid	13.10 ± 1.94	11.97 ± 5.16	12.39 ± 2.68
Ethylmalonic Acid	17.48 ± 0.00	17.48 ± 0.00	17.48 ± 0.00
Succinic Acid	25.73 ± 6.65	29.57 ± 8.97	35.31 ± 6.55
Fumaric Acid	0.01 ± 0.00	0.01 ± 0.00	0.01 ± 0.00
Glutaric Acid	0.81 ± 0.00	0.80 ± 0.00	0.80 ± 0.00
Capric Acid	8.93 ± 0.51	8.75 ± 0.99	9.02 ± 0.90
Oxaloacetic Acid	1.57 ± 0.03	1.53 ± 0.01	1.53 ± 0.01
Malic Acid	47.61 ± 12.20	42.29 ± 11.40	54.50 ± 40.94
Adipic Acid	0.13 ± 0.00	0.14 ± 0.00	0.14 ± 0.00
Pyroglutamic Acid	153.32 ± 26.24	100.33 ± 17.30	221.38 ± 47.05
Pimelic Acid	0.28 ± 0.01	0.27 ± 0.00	0.28 ± 0.01
α-Ketoglutaric Acid	1.12 ± 0.24	1.10 ± 0.20	2.27 ± 1.35
PPAMS	2.19 ± 0.00	2.19 ± 0.00	2.53 ± 0.92
Suberic Acid	0.51 ± 0.00	0.51 ± 0.00	0.52 ± 0.00
Orotic Acid	0.25 ± 0.04	0.20 ± 0.06	0.31 ± 0.05
cis-Aconitic Acid	0.09 ± 0.00	0.10 ± 0.00	0.10 ± 0.00
Citric Acid	27.17 ± 8.60	28.79 ± 7.28	36.90 ± 15.41
Isocitric Acid	0.47 ± 0.00	0.47 ± 0.01	0.48 ± 0.01
Sebacic Acid	0.17 ± 0.00	0.18 ± 0.00	0.18 ± 0.00

2.2 FFA profiles by GC-MS

TABLE S2 | The FFA profiles of 90 subjects.

FFA (μg/mL)	NC (n = 30)	GDM (n = 30)	HP (n = 30)
C14:0	6.71 ± 2.95	8.06 ± 3.52	8.78 ± 4.02
C16:0	364.81 ± 85.42	416.44 ± 66.01	482.28 ± 152.43
C16:1	14.22 ± 4.39	18.49 ± 4.48	20.38 ± 5.94
C18:0	121.91 ± 32.17	132.88 ± 23.23	149.75 ± 46.36
C18:1	174.07 ± 48.36	216.01 ± 51.12	255.98 ± 59.98
C18:2	184.44 ± 42.16	167.93 ± 25.41	174.42 ± 33.78
C18:3γ	1.65 ± 0.71	1.41 ± 0.52	1.37 ± 0.47
C18:3α	11.76 ± 5.21	8.84 ± 2.70	8.90 ± 4.38
C18:4	0.32 ± 0.23	0.30 ± 0.19	0.32 ± 0.14
C20:2	0.44 ± 0.19	0.30 ± 0.10	0.32 ± 0.14
C20:3	28.32 ± 5.97	26.20 ± 7.84	28.57 ± 9.65
C20:4	205.59 ± 44.03	174.57 ± 35.31	196.19 ± 45.89
C20:5	32.91 ± 19.89	31.38 ± 17.55	32.15 ± 12.86
C22:4	0.62 ± 0.44	0.50 ± 0.26	0.45 ± 0.11
C22:5	2.77 ± 1.12	2.18 ± 0.81	2.49 ± 1.32
C22:6	26.16 ± 6.20	20.50 ± 6.23	21.82 ± 5.35

2.3 AA profiles by UPLC-TQ-MS

TABLE S3 | The AA profiles of 90 subjects.

AA (mM)	NC (n = 30)	GDM (n = 30)	HP (n = 30)
Threonine	232.28 ± 47.27	224.27 ± 68.95	291.82 ± 103.68
Glutamine	258.51 ± 37.00	248.90 ± 37.13	308.94 ± 116.57
Leucine	25.17 ± 8.18	30.19 ± 7.15	27.46 ± 7.85
Arginine	108.49 ± 17.56	113.61 ± 24.10	103.64 ± 16.85
Valine	155.39 ± 38.20	174.27 ± 26.29	166.09 ± 47.93
Isoleucine	33.61 ± 10.09	40.34 ± 11.51	35.05 ± 9.51
Phenylalanine	9.51 ± 1.80	10.94 ± 2.15	11.49 ± 3.06
Tryptophan	25.20 ± 5.15	23.62 ± 5.42	23.98 ± 7.63
Serine	199.06 ± 45.09	203.24 ± 36.72	225.42 ± 47.13
Methionine	11.29 ± 2.53	11.37 ± 3.01	13.55 ± 5.54
Glycine	159.84 ± 52.52	187.25 ± 38.50	199.33 ± 73.26
Proline	117.12 ± 23.96	118.62 ± 28.56	111.49 ± 26.55
Histidine	124.30 ± 21.26	120.49 ± 16.45	131.53 ± 22.23
Alanine	389.80 ± 105.46	368.70 ± 78.03	411.90 ± 118.65
Lysine	179.56 ± 39.77	192.00 ± 46.97	188.97 ± 55.53
γ-AminobutyricAcid	1224.9 ± 202.74	1208.18 ± 184.61	1663.94 ± 402.75
Creatinine	59.50 ± 19.12	49.54 ± 8.77	73.17 ± 23.14
Dimethylglycine	3.90 ± 1.22	4.43 ± 1.07	5.20 ± 2.03
Creatine	29.63 ± 8.74	27.55 ± 8.34	29.03 ± 14.34
GlutamicAcid	103.18 ± 20.45	125.24 ± 33.69	136.9 ± 41.16
Taurine	515.87 ± 135.80	493.48 ± 131.02	465.97 ± 135.72
Asparagine	42.83 ± 8.77	38.83 ± 6.17	43.01 ± 13.18
Tyrosine	32.52 ± 10.92	39.12 ± 10.41	37.43 ± 15.68
AminbutyricAcid	1.62 ± 0.49	1.78 ± 0.40	1.88 ± 0.68
Acetylcarnitine	4.46 ± 2.25	4.47 ± 1.69	6.73 ± 3.23
Ornithine	49.35 ± 13.31	45.51 ± 9.81	55.49 ± 18.54
Citrulline	10.46 ± 2.80	8.69 ± 2.24	10.90 ± 2.14

3 WGCNA analysis

3.1 Method of WGCNA Analysis

In the study, 27 amino acids, 16 fatty acids, and 29 organic acids were measured in serum. We first calculated Pearson's correlation coefficient between the expression amounts of metabolites after basic processing, such as the detection of missing values and abnormal points. The expression data was converted to a correlation matrix.

Subsequently, an adjacency matrix was created by importing the power exponent weighting and applying the N power of the metabolite correlation coefficient. The connections between the metabolites in the network obeyed the scale-free topology model (SFT). Furthermore, SFT model transformation should satisfy the following criteria: (1) fit indices, signed $R^2 > 0.8$; (2) model slope ~ -1; and (3) higher mean data connectivity.

Thirdly, the metabolites with a high distance of topological overlap (distTOM) were gathered into one module by the method of average linkage hierarchical clustering. Through multiple transformations of three matrices, metabolites with similar expression patterns were assembled into one module.

Finally, the correlations between module and phenotype data of the samples were calculated, and the interaction network linkages between the metabolites contained in specific modules were examined. The 12 types of algorithms in the cytoHubba plug-in of Cytoscape (version 3.8.2) were used to extract hub metabolites with a high degree of connectivity in the correlation expression network (the top 5% were extracted).

3.2 Results of WGCNA Analysis between NC and HP

The SFT model in WGCNA analysis was conducted with soft thresholding power set at 3 **(Figure 1A)**. WGCNA illustrated 72 metabolites were assembled into 5 modules **(Figure 1B)**. We then calculated correlation between 5 modules and traits referring to HP, and found turquoise module was significantly associated with SBP, DBP and HP, and even the correlation coefficients were close (MEturquoise ~ HP, r = 0.41, $p < 0.05$) **(Figure 1C)**, suggesting the potential confounding effects of clinical parameters on the serum metabolite levels. In addition, the hub metabolites within turquoise module with high intramodular importance were identified by 12 types of algorithms in the cytoHubba plug-in of Cytoscape, which included GABA, glutamic acid and pyroglutamic acid. Moreover, we made an interesting discovery that most metabolites in the turquoise module were amino acids, which indicated that amino acids play a more important role in the development of HP, compared with fatty acids and organic acids **(Figure 1D)**. These findings across WGCNA analysis were in accordance with multivariate analysis and RF analysis in the manuscript.

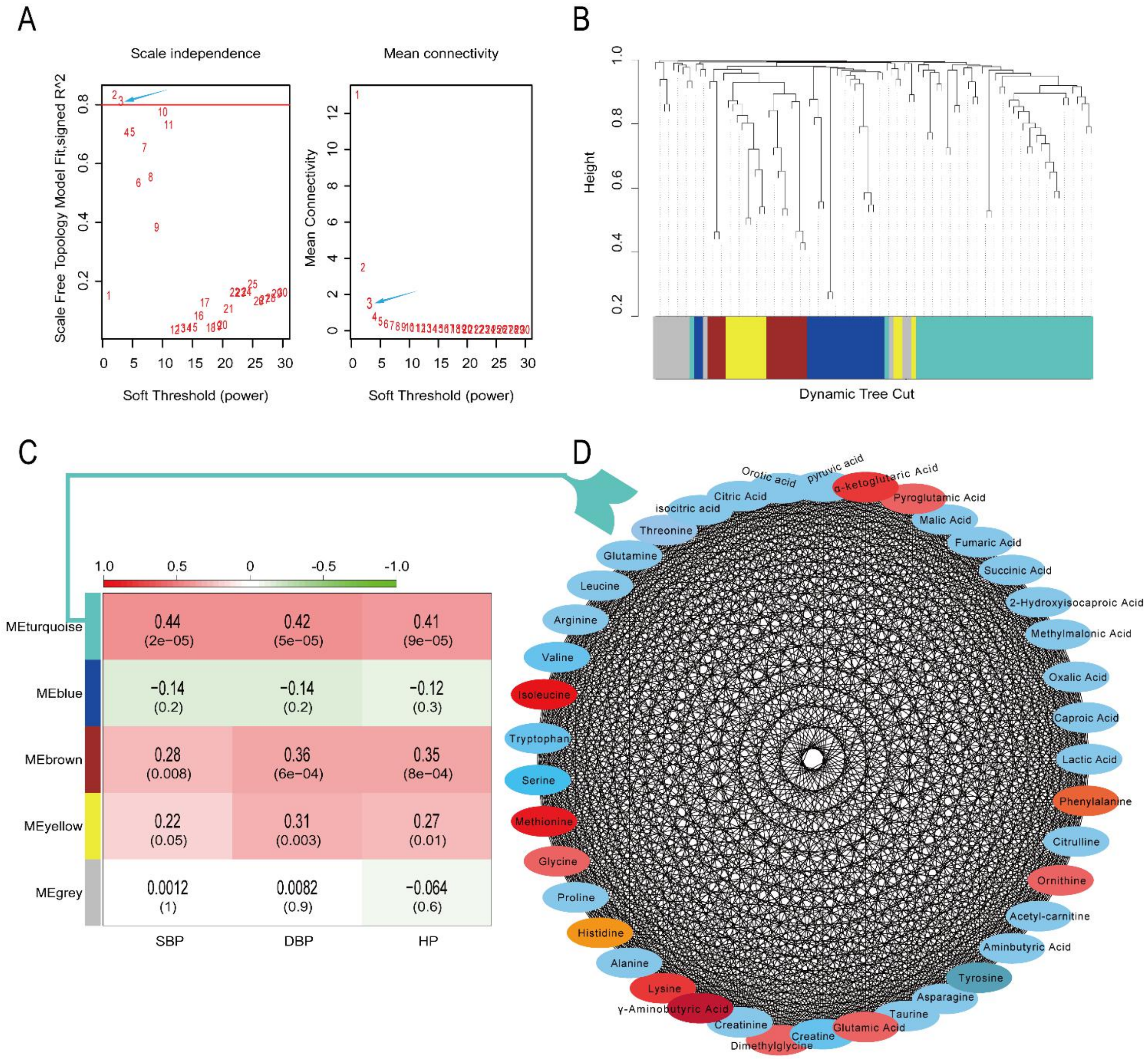

FIGURE 1 | Identification of remarkable metabolites by WGCNA analysis. **(A)** Soft thresholding power and mean connectivity by SFT model. **(B)** Dendrogram showing metabolite co-expression modules defined in 90 samples. **(C)** Pearson's correlation coefficient between SBP, DBP, HP and five distinct modules. **(D)** Co-expression network of hub metabolites within turquoise module.

4 Dietary data by FFQ

In this study, all participants completed a validated, internet-based food-frequency and lifestyle questionnaire (www.yyjy365.org/diet). The reproducibility and validity of Food Frequency Questionnaire (FFQ) were assessed in a previous study **(1, 2)**. The FFQ for pregnant women was mainly used to investigate the frequency and amount of food intake for a period of time since the middle of pregnancy (from 14 weeks of pregnancy). The FFQ contains 17 categories of food (Cereals,

potatoes, beans, vegetables, fungi and algae, fresh fruits, nut seeds, poultry, livestock, milk, eggs, fish, instant foods, sugars, beverages, seasonings and others) and 143 specific food items. The intakes of calorie and nutrients of each pregnant woman were calculated according to the Chinese Food Composition Tables (6th edition).

4.1 Calculation of the major productive nutrients - energy ratio of fat, carbohydrate and protein are as follows:

$$\frac{\text{Fat intake(g/d)} * 9(kcal/g)}{Total\ energy\ intake(kcal/d)} * 100\%$$

$$\frac{\text{Carbohydrate intake(g/d)} * 4(kcal/g)}{Total\ energy\ intake(kcal/d)} * 100\%$$

$$\frac{\text{Protein intake(g/d)} * 4(kcal/g)}{Total\ energy\ intake(kcal/d)} * 100\%$$

4.2 Raw data referring to dietary energy using a FFQ

TABLE S4 | The dietary levels of fat, carbohydrate and protein intake in 90 participants

ID number	Fat intake（g/d）	Carbohydrate intake（g/d）	Protein intake（g/d）	Total energy intake（kcal/d）	Fat-Total energy ratio（%）	Carbohydrate-Total energy ratio（%）	Protein-Total energy ratio（%）
NC1	95.94	381.14	70.43	2669.79	0.32	0.57	0.11
NC2	39.04	810.70	43.96	3769.95	0.09	0.86	0.05
NC3	119.33	760.51	263.19	5168.76	0.21	0.59	0.20
NC4	166.23	375.24	94.51	3375.03	0.44	0.44	0.11
NC5	20.85	505.99	97.75	2602.60	0.07	0.78	0.15
NC6	42.46	398.07	62.81	2225.67	0.17	0.72	0.11
NC7	48.93	272.56	57.41	1760.22	0.25	0.62	0.13
NC8	66.28	247.55	77.31	1895.93	0.31	0.52	0.16
NC9	60.57	386.03	91.57	2455.49	0.22	0.63	0.15
NC10	103.29	458.20	23.14	2854.96	0.33	0.64	0.03
NC11	42.78	273.94	95.24	1861.70	0.21	0.59	0.20
NC12	113.82	362.40	46.29	2659.08	0.39	0.55	0.07
NC13	35.71	471.21	97.90	2597.82	0.12	0.73	0.15
NC14	39.40	559.75	62.88	2845.13	0.12	0.79	0.09
NC15	41.94	258.86	153.53	2027.02	0.19	0.51	0.30
NC16	159.92	228.05	94.42	2729.13	0.53	0.33	0.14
NC17	44.77	445.11	201.64	2989.90	0.13	0.60	0.27
NC18	56.58	776.30	116.38	4080.00	0.12	0.76	0.11
NC19	140.87	512.23	59.96	3556.62	0.36	0.58	0.07
NC20	112.38	592.43	82.63	3711.69	0.27	0.64	0.09
NC21	75.91	505.89	59.94	2946.54	0.23	0.69	0.08
NC22	83.24	568.64	78.06	3335.98	0.22	0.68	0.09
NC23	42.41	300.93	37.13	1733.91	0.22	0.69	0.09

NC24	62.65	276.63	90.60	2032.78	0.28	0.54	0.18
NC25	68.84	404.99	103.17	2652.23	0.23	0.61	0.16
NC26	64.13	687.56	95.06	3707.62	0.16	0.74	0.10
NC27	88.76	385.97	57.28	2571.85	0.31	0.60	0.09
NC28	127.32	315.35	117.26	2876.28	0.40	0.44	0.16
NC29	111.23	355.75	63.03	2676.21	0.37	0.53	0.09
NC30	28.57	644.51	91.48	3201.07	0.08	0.81	0.11
GDM1	74.78	312.01	64.94	2180.81	0.31	0.57	0.12
GDM2	91.49	447.57	140.27	3174.76	0.26	0.56	0.18
GDM3	82.39	403.82	76.60	2663.25	0.28	0.61	0.12
GDM4	99.76	530.91	133.69	3556.27	0.25	0.60	0.15
GDM5	72.84	384.78	93.83	2570.00	0.26	0.60	0.15
GDM6	237.66	294.89	160.06	3958.77	0.54	0.30	0.16
GDM7	81.67	1078.72	94.56	5428.11	0.14	0.79	0.07
GDM8	153.57	235.50	109.84	2763.51	0.50	0.34	0.16
GDM9	108.11	504.60	107.26	3420.44	0.28	0.59	0.13
GDM10	174.05	323.30	108.96	3295.50	0.48	0.39	0.13
GDM11	229.63	326.68	64.22	3630.23	0.57	0.36	0.07
GDM12	101.52	444.85	165.85	3356.46	0.27	0.53	0.20
GDM13	93.46	887.72	119.47	4869.91	0.17	0.73	0.10
GDM14	199.76	333.54	114.09	3588.37	0.50	0.37	0.13
GDM15	131.93	282.82	113.45	2772.45	0.43	0.41	0.16
GDM16	153.76	520.86	121.42	3952.89	0.35	0.53	0.12
GDM17	214.90	604.90	94.05	4729.85	0.41	0.51	0.08
GDM18	200.50	424.54	56.81	3729.97	0.48	0.46	0.06
GDM19	170.14	521.43	109.40	4054.60	0.38	0.51	0.11
GDM20	119.46	386.87	140.21	3183.48	0.34	0.49	0.18
GDM21	85.28	896.93	94.60	4733.60	0.16	0.76	0.08
GDM22	63.63	476.50	124.49	2976.65	0.19	0.64	0.17
GDM23	135.82	416.36	139.74	3446.75	0.35	0.48	0.16
GDM24	98.82	834.09	116.18	4690.45	0.19	0.71	0.10
GDM25	104.31	505.90	88.93	3318.16	0.28	0.61	0.11
GDM26	158.04	639.53	83.33	4313.82	0.33	0.59	0.08
GDM27	57.65	760.30	97.30	3949.19	0.13	0.77	0.10
GDM28	142.61	754.86	82.48	4632.85	0.28	0.65	0.07
GDM29	130.01	657.00	35.88	3941.60	0.30	0.67	0.04
GDM30	103.18	668.07	105.37	4022.32	0.23	0.66	0.10
HP1	108.48	511.48	115.09	3482.60	0.28	0.59	0.13
HP2	101.88	500.03	99.50	3315.05	0.28	0.60	0.12
HP3	196.47	428.33	49.52	3679.63	0.48	0.47	0.05
HP4	80.78	445.25	152.91	3119.70	0.23	0.57	0.20
HP5	201.10	490.01	81.24	4094.92	0.44	0.48	0.08
HP6	70.87	472.86	74.93	2828.97	0.23	0.67	0.11
HP7	61.82	479.28	126.87	2981.03	0.19	0.64	0.17
HP8	103.65	466.38	125.09	3298.68	0.28	0.57	0.15
HP9	93.49	496.08	152.60	3436.11	0.24	0.58	0.18
HP10	67.10	514.84	103.48	3077.18	0.20	0.67	0.13

HP11	66.23	468.82	154.77	3090.37	0.19	0.61	0.20
HP12	120.59	543.33	115.49	3720.61	0.29	0.58	0.12
HP13	55.82	998.19	38.42	4648.80	0.11	0.86	0.03
HP14	40.36	467.41	62.50	2482.92	0.15	0.75	0.10
HP15	77.40	448.99	76.07	2796.83	0.25	0.64	0.11
HP16	61.37	430.39	68.89	2549.46	0.22	0.68	0.11
HP17	39.68	525.67	77.22	2768.74	0.13	0.76	0.11
HP18	111.31	450.68	128.37	3318.00	0.30	0.54	0.15
HP19	95.43	490.56	97.74	3212.08	0.27	0.61	0.12
HP20	115.58	463.62	122.16	3383.36	0.31	0.55	0.14
HP21	117.11	521.01	103.30	3551.20	0.30	0.59	0.12
HP22	33.80	537.70	60.70	2697.75	0.11	0.80	0.09
HP23	61.79	497.47	146.98	3133.89	0.18	0.63	0.19
HP24	63.16	483.63	128.86	3018.43	0.19	0.64	0.17
HP25	85.56	627.21	36.31	3424.10	0.22	0.73	0.04
HP26	146.43	498.64	93.88	3687.95	0.36	0.54	0.10
HP27	88.36	494.68	126.24	3278.87	0.24	0.60	0.15
HP28	72.76	453.94	129.30	2987.78	0.22	0.61	0.17
HP29	105.03	442.57	117.01	3183.61	0.30	0.56	0.15
HP30	139.54	563.96	100.45	3913.48	0.32	0.58	0.10

5 Binary logistic regression for NC-GDM model and NC-HP model.

TABLE S5 | Binary logistic regression for NC-GDM model and NC-HP model.

Models	Factors	OR	95%CI	*p*-Value
NC vs GDM model	BMI	1.709	0.962-3.036	0.068
	Pyroglutamic Acid	0.824	0.720-0.944	**0.005**
	2-Hydroxybutyric Acid	1.013	0.971-1.056	0.552
	Glutamic Acid	1.051	0.987-1.12	0.121
NC vs HP model	BMI	4.419	0.673-29.015	0.122
	γ-Aminobutyric Acid	1.016	1.000-1.033	**0.048**
	Glutamic Acid	1.094	0.977-1.225	0.119
	Pyroglutamic Acid	1.139	1.014-1.279	**0.028**
	C16:0	1.016	0.993-1.040	0.162

Referrence

[1] Cheng Y, Zhang K, Chen Y, et al. Associations between Dietary Nutrient Intakes and Hepatic Lipid Contents in NAFLD Patients Quantified by ^{1}H-MRS and Dual-Echo MRI. Nutrients. 2016;8(9):527.

[2] Yang C, Rennan F, Xue Y, et al. Yogurt improves insulin resistance and liver fat in obese women with nonalcoholic fatty liver disease and metabolic syndrome: a randomized controlled trial. Am J Clin Nutr, 2019 1;109(6):1611-1619.
